# Socioemotional Skills Program with a Group of Socioeconomically Disadvantaged Young Adolescents: Impacts on Self-Concept and Emotional and Behavioral Problems

**DOI:** 10.3390/children9050680

**Published:** 2022-05-07

**Authors:** Lurdes Veríssimo, Isabel Castro, Marisa Costa, Pedro Dias, Francisca Miranda

**Affiliations:** 1 CEDH—Research Centre for Human Development, Universidade Católica Portuguesa, 4169-005 Porto, Portugal; mrcosta@ucp.pt (M.C.); pmbdias@ucp.pt (P.D.); 2 Faculty of Education and Psychology, Universidade Católica Portuguesa, 4169-005 Porto, Portugal; icastro@ucp.pt (I.C.); fmiranda@ucp.pt (F.M.); 3 Department of Psychology, University of the Azores, 9500-321 Ponta Delgada, Portugal

**Keywords:** socioemotional skills, self-concept, emotional and behavioral problems, intervention effectiveness, early adolescents

## Abstract

There is significant evidence that emphasizes the importance of social and emotional learning in schools for students’ positive development and adjustment. The main goal of the present study was to evaluate the effectiveness of a socioemotional skills promotion program, implemented with a group of socioculturally vulnerable young adolescents. Data were collected in the 2020–2021 school year from all students from 6th grade (*n* = 50, from four classes) in a high-risk school in Portugal (56% females). Two classes served as the comparison group. Participants responded to self-concept and emotional and behavioral problems measures at two moments (pre- and post-intervention). Results indicated a significant impact on self-concept, namely an increase in behavioral adjustment, happiness, and satisfaction and a decrease in anxiety in the intervention group compared with the comparison group. These findings support intervention efficacy. Educational implications are discussed.

## 1. Introduction

Socioemotional learning (SEL) is an extremely important process in children’s and adolescents’ educational trajectory. SEL allows the acquisition and the effective use of the knowledge, attitudes, and skills necessary to understand, manage, and express emotions in an adjusted way, to establish and achieve goals, to feel and express empathy for others, to establish and maintain interpersonal relationships, and to make responsible and informed decisions. The most used framework for SEL—collaborative for academic and social and emotional learning (CASEL)—has identified five core social and emotional competences clusters: self-awareness, self-management, social awareness, relationship skills, and responsible decision-making [1].

Research evidence is consensual to show that socioemotional development is considered one of the predictors of positive outcomes both at school (e.g., academic achievement, and positive relationships with peers) and throughout life (e.g., social inclusion or integration into the labor market) [2,3]. Empirical evidence confirms that the implementation of SEL programs allows: (1) students’ greater attitudinal adjustment and higher ability to effectively manage their difficulties; (2) increased academic achievement; (3) a reduction of emotional and behavioral problems and psychological distress; and (4) the promotion of more positive school environments, with a lower prevalence of bullying behaviors [4,5,6,7,8]. In particular, the promotion of socioemotional development, specifically the stimulation of skills related to self-control, empathy, and problem solving are associated with higher levels of motivation and school involvement, therefore enhancing academic success and preventing students from dropping out of school [9,10,11,12]. Furthermore, the lack of stimulation of socioemotional skills contributes negatively to the academic success of students: students who express gaps in their socioemotional development tend to reveal low school involvement and achievement, higher dropout rates [10], and greater difficulties in behavioral adjustment [13,14]. Kaşıkcı and Özhan [15], in a study involving 337 students aged between 10 and 14 years, found a significant positive correlation among socioemotional learning and academic performance and levels of happiness. In the same study, socioemotional learning was a significant predictor of academic performance. Coelho et al. [16] evaluated the effectiveness of a socioemotional development program in 1063 Portuguese students aged between 8 and 11 years. Students who participated in this program showed significant improvement in terms of social awareness, self-control, and self-esteem. 

In the specific case of children and young people from disadvantaged and multi-challenged socioeconomic contexts in which the most basic dimensions of socioemotional development, such as self-concept, are often quite low, the development of socioemotional skills may represent an important protective factor [13].

The present study aims to evaluate the effectiveness of a specific program to promote socioemotional development in a high-risk sample from a multi-challenged school in Portugal.

## 2. Materials and Methods

### 2.1. Participants

The study included 50 participants from 4 classes of the 6th grade (56% female) with a mean age of 11.38 years (SD = 0.80, range 10–13 years). Participants attended a high-risk public school. The comparison group (CG) included 27 participants (2 classes) and an intervention group (IG) of 23 students (2 classes).

### 2.2. Measures

#### 2.2.1. Youth Self-Report (YSR) 

The YSR [17,18] is a self-report measure for the assessment of emotional and behavioral problems in adolescents aged between 11 and 18 years-old. It includes 112 items, organized in 8 syndromes, which are organized in the second-order scales of Internalizing Problems (e.g., “I cry a lot”), Externalizing Problems (e.g., “I physically attack people”), and Total Problems.

Participants were requested to rate their functioning over the last 6 months using a Likert scale anchored with the descriptors: 0 (not true), 1 (somehow or sometimes true) and 2 (very true or often true). The psychometric properties of YSR were assessed and revealed adequate [18]. In the Portuguese normative sample, the YSR alpha coefficient was α = 0.93 for Total Problems, α = 0.84 for Internalizing Problems, and α = 0.85 for Externalizing Problems.

#### 2.2.2. Piers-Harris Children’s Self-Concept Scale (PHCSCS-2)

The PHCSCS-2 [19,20] assesses self-concept in six dimensions: Behavioral Adjustment (BA) (e.g., “I am well behaved in school”), Intellectual and School Status (ISS) (e.g., “I am smart”), Physical Appearance and Attributes (PAA) (e.g., “I have nice hair”), Anxiety (AN) (e.g., “I am often afraid”), Popularity (PO) (e.g., “I have many friends”), and Happiness and Satisfaction (SF) (e.g., “I am cheerful”) [20]. Internal consistency values were adequate for all the scales: Behavioral Adjustment, α = 0.74; Intellectual and School Status, α = 0.75; Physical Appearance and Attributes, α = 0.72; Anxiety, α = 0.62; Popularity, α = 0.70; Happiness and Satisfaction, α = 0.67; Total Scale, α = 0.85.

### 2.3. The Intervention Program

The intervention program was implemented as part of a broader project called Learning with All, whose main goal was to promote school success and prevent early school dropout. The project was implemented in a medium-sized public-school cluster in Portugal. This school was inserted in a disadvantaged urban community with social and economic hardships. The students presented high levels of absenteeism and behavioral problems as well as low levels of academic achievement.

The program was implemented by the psychologists of the Learning with All Project with 6th grade students over 12 sessions. The selection of this sample was based on the needs reported by the teachers. All the students attending the 6th grade were included in the study.

The intervention was implemented during school hours, 1 h per week, over a 5-month period, from November 2020 to May 2021. Due to restrictions related to the COVID-19 pandemic, the program was suspended for 10 weeks (from late January to the second week of April 2021).

Prior to implementation, the research team met with the teachers of each class to assess the main students’ difficulties in socioemotional development in order to select the most relevant content for the intervention program. Based on this needs evaluation, the program was designed to achieve the following main goals: (1) to promote self-concept and self-esteem; (2) to stimulate emotional management and self-control (e.g., behavioral self-regulation, impulse control, cognitive restructuring of irrational beliefs, anxiety management); and (3) to train assertiveness. Conceptually, the program was based on the background of CASEL [1,21], and on the multi-tiered systems of support (MTSS) approach [21]. Furthermore, the program adopted the SAFE methodology recommended by CASEL: sequential, active, focused and explicit [4,21]. Therefore, the activities followed a sequential training approach, using active forms of learning (e.g., short videos, dilemma-based stories, interactive activities, and group discussion).

The students’ native language was Portuguese. The survey and the intervention program were both administered in this language. For a detailed description of the intervention program, see Veríssimo et al., in press [22].

### 2.4. Procedures

The data collection procedure complied with legal and ethical requirements. First, authorization was given by the direction/pedagogical board of the school. Parents received information about the study goals as well as the procedures, and their written consent was obtained. Students’ anonymity was assured by using codes for each participant. 

Data collection occurred in two moments, previous (pre-intervention), in November 2020 and immediately after the intervention (post-intervention), in May 2021. Students took approximately 40 min filling out the assessment protocol. The intervention and the comparison groups, based on pre-existing classes, were equally and homogeneously distributed considering the pre-intervention results. After the post-intervention assessment, the program was delivered to the participants from the comparison group.

### 2.5. Statistical Analysis

To analyze the intra- and inter-individual evolution of students’ self-concept and emotional and behavioral problems, an analysis of variance for repeated measures (GLM) was conducted. The factors considered were time (intra-subject factor) and group (inter-subject factor).

First, the assumptions for repeated measures were verified and ensured, namely the normality of the sample distribution in the different variables [23]. All the dimensions of self-concept and emotional and behavioral problems were normally distributed, according to the Shapiro–Wilk test (*p* > 0.10 for the 2 moments). Other assumptions for repeated measures referred to the homogeneity of the covariance matrix and sphericity, evaluated using the Box M test and the Mauchly sphericity test, respectively [23], which presented the value of *p* > 0.05 in all analyses, thus confirming these assumptions.

## 3. Results

**Figure 1:**
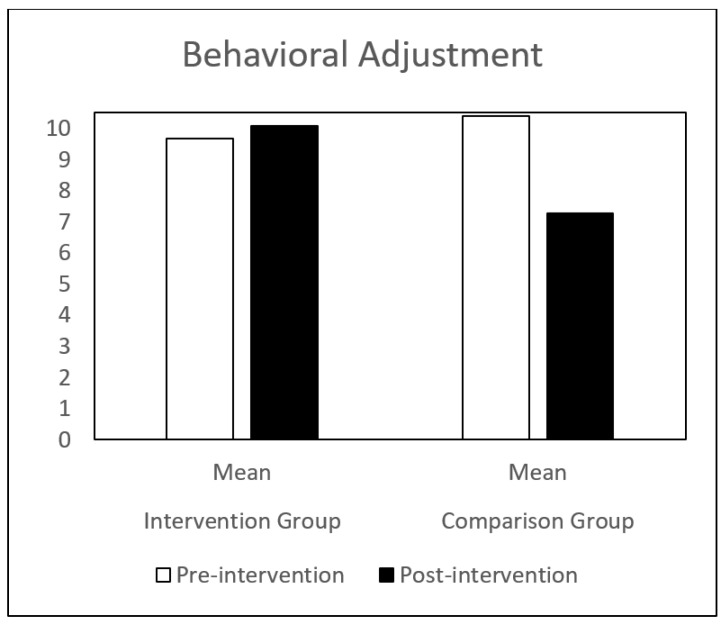
Behavioral Adjustment mean in IG and CG at pre- and post-intervention.

**Figure 2:**
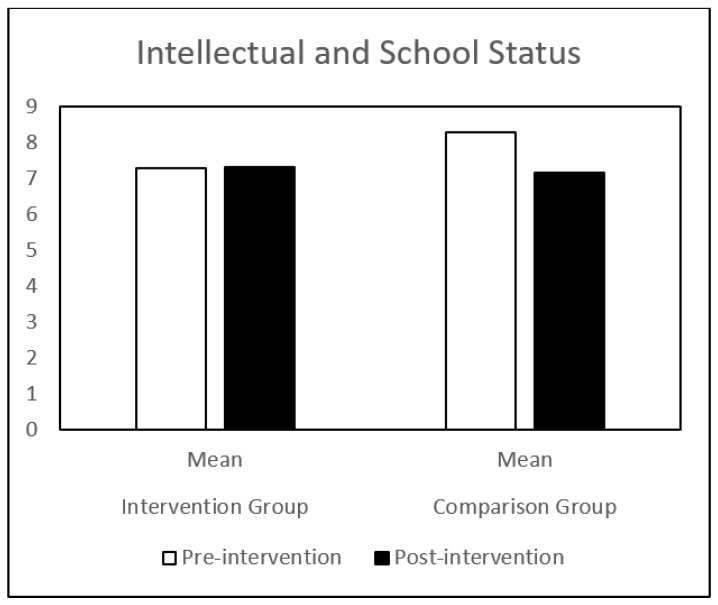
Intellectual and School Status mean in IG and CG at pre- and post-intervention.

**Figure 3:**
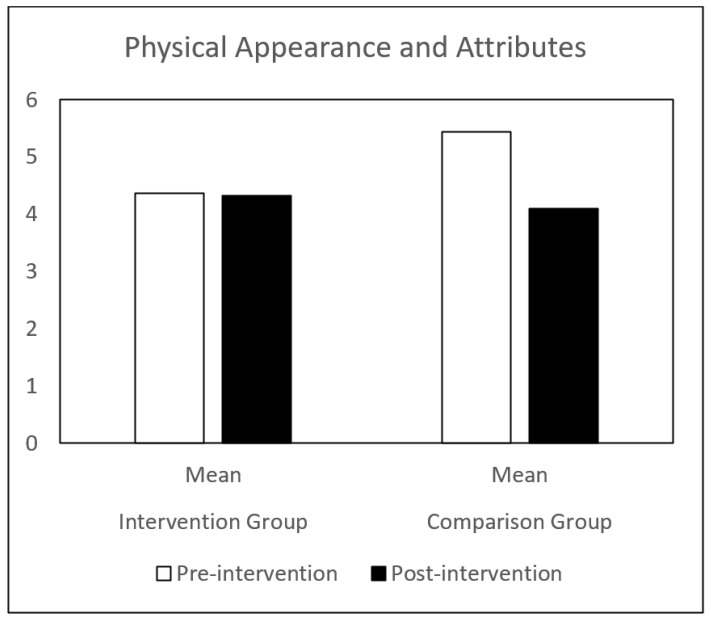
Physical Appearance and intervention. Intervention Attributes mean in IG and CG at pre- and post-intervention.

**Figure 4:**
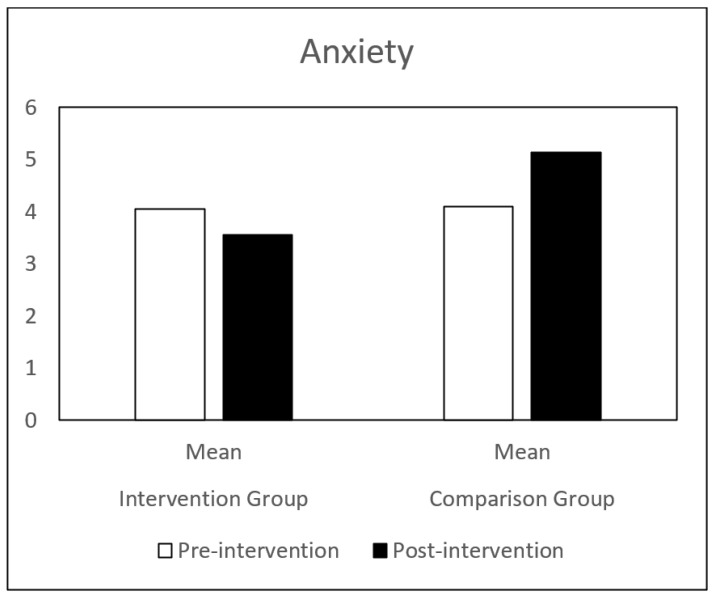
Anxiety mean in IG and CG at pre- and post-intervention.

**Figure 5:**
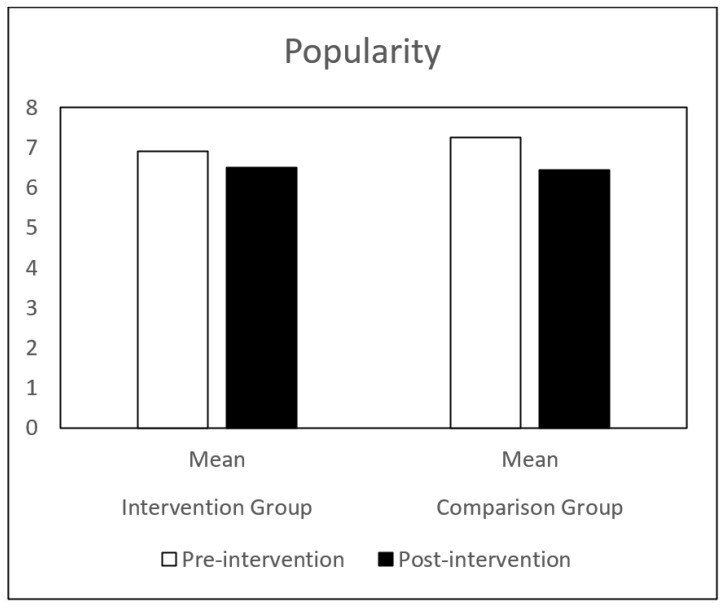
Popularity mean in IG and CG at pre- and post-intervention.

**Figure 6:**
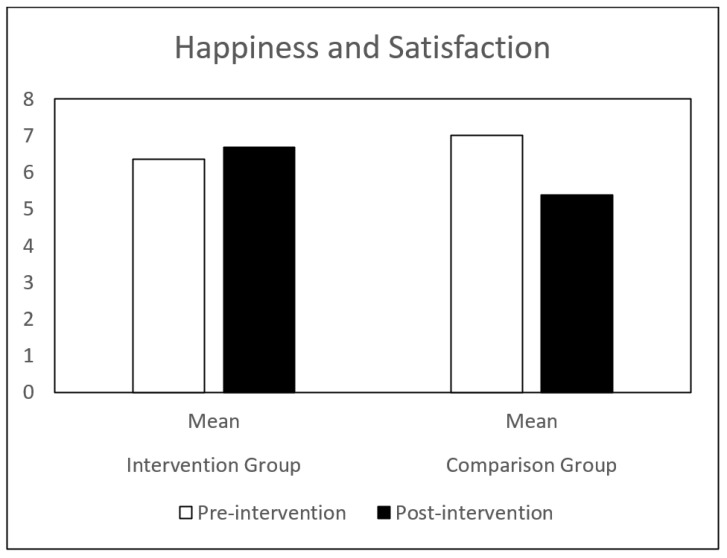
Happiness and Satisfaction mean in IG and CG at pre- and post-intervention.

**Figure 7:**
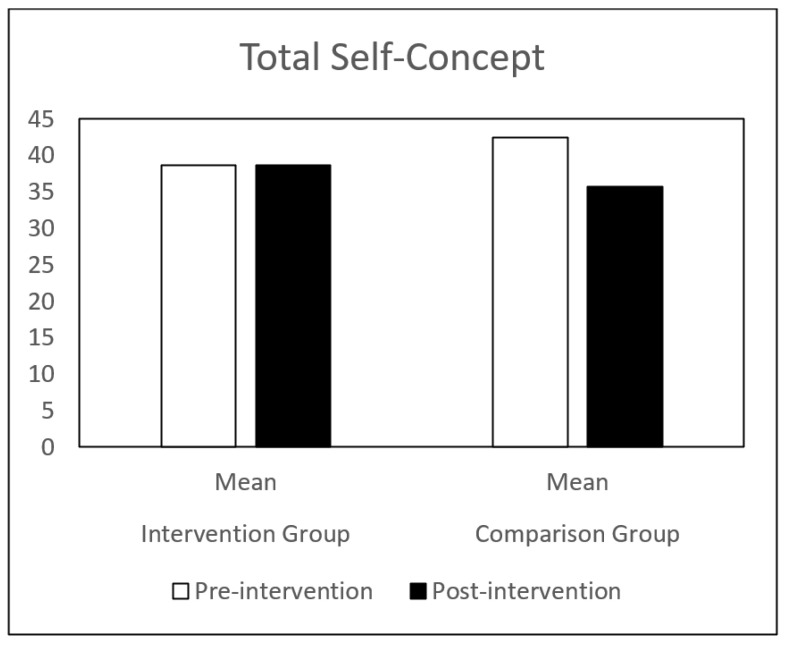
Total Self-concept mean in IG and CG at pre- and post-intervention.

Regarding self-concept, both groups (IG and CG) presented similar results at pre-intervention. Globally, the CG showed a decrease in all the self-concept dimensions, between pre-intervention and post-intervention. The IG showed a significant increase in behavioral adjustment and happiness and satisfaction, and a significant decrease in anxiety between the same time points. There is a significant difference in the evolution of these dimensions between the IG and the CG groups (Table 1 and Figure 1, Figure 2, Figure 3, Figure 4, Figure 5, Figure 6 and Figure 7). 

The repeated-measures GLM showed a significant interaction effect in behavioral adjustment (Z (1.43) = 8.66, *p* < 0.005, η^2^ = 0.17), happiness and satisfaction (Z (1.43) = 5.94, *p* < 0.019, η^2^ = 0.12), and Anxiety (Z (1.43) = 4.42, *p* < 0.041, η^2^ = 0.09). No significant interaction effect was found in the other self-concept dimensions (intellectual and school status, physical appearance and attributes, popularity, and total self-concept).

The comparison of emotional and behavioral problems between groups and moments (pre- and post-intervention) did not reveal significant effects (Table 2).

## 4. Discussion and Conclusions

This study aimed to evaluate the effectiveness of a tailored intervention program focused on the development of socioemotional skills in a group of sixth graders from a high-risk school context. The overall results show that the intervention had no significant impact on emotional and behavioral problems. However, positive and significant changes in three dimensions of self-concept—behavioral adjustment, anxiety, and happiness and satisfaction—were shown by participants from the intervention group, whereas the participants from the comparison group revealed reduced scores in these dimensions of self-concept from pre- to post-intervention. These results are consistent with previous studies showing a positive impact of SEL in self-control and self-esteem in normative [16] and in socioculturally vulnerable groups [13]. Examining the different dimensions of the self-concept measure, the fact that no significant impact was found in intellectual and school status, physical appearance and attributes, and popularity could be explained by the specific goals and syllabus of the intervention program. On the one hand, the program does not focus on intellectual and school status, physical dimensions, and popularity; on the other hand, behavioral control, anxiety, and general wellbeing were deeply addressed during the sessions. Overall, the significant results concerning self-concept are encouraging since these kinds of competencies are crucial for well-adjusted psychological developmental trajectories [4].

The absence of a significant impact of the program on emotional and behavioral problems could be explained through different perspectives. First, when compared with the Portuguese normative samples of adolescents, participants from both groups in the present study showed baseline high levels of internalizing, externalizing, and total problems in the YSR, in line with studies conducted with other vulnerable samples [24]. These participants come from a context continuously exposed to environmental risk factors, such as domestic violence, poverty, and family dysfunction. The intervention program did not focus on these dimensions that could affect the emotional and the behavioral problems’ levels. Second, participants from the intervention group probably became more aware of their psychological functioning (e.g., through sessions focused on self-awareness or emotion recognition). Therefore, the non-expected maintenance of self-reported emotional and behavioral problems in the intervention group could translate to an increase in the self-demanding evaluation of their own symptoms. Finally, the post intervention assessment occurred approximately one month after the end of a long lockdown due to COVID-19. Thus, the decrease in emotional and behavioral problems in the comparison group may reflect an increase in wellbeing after months of lockdown. 

Although the results did not show, as expected, a positive impact of the program in all assessed dimensions, we can assume that this intervention induced positive changes, and it could be implemented with vulnerable young adolescents.

One of this study’s limitation was the exclusive use of self-report measures. Future studies should include other informants (e.g., teacher’s perceptions of emotional and behavioral problems) to have a broader evaluation of the intervention’s impact. A second limitation is related to the sample used in the study—all students came from the same school, considering the larger project where it was anchored—and the sample size was modest. This limitation hindered the use of more sophisticated statistical analyses that would allow a deeper understanding of the effects of the intervention. Thus, it is important to replicate this study with larger samples from different school contexts and to use different and more robust statistical analyses. As mentioned above, the implementation of the intervention program was interrupted due to COVID-19 restrictions, which directly affected the operationalization of the program. Furthermore, the psychosocial consequences of the pandemic [25,26] probably interfered with the key variables of the study (self-concept and emotional and behavioral problems). Therefore, future research with this program would benefit from its implementation during a period without the constraints of the pandemic.

School represents a relevant context for SEL [27,28], strongly contributing to the satisfaction of students’ basic psychological needs [29]. In this sense, SEL programs incorporation, based on MTSS should provide universal services, directed to all students, considering their specific characteristics and needs [30,31,32], ensuring mental health and academic success in all children and adolescents [15,33].

## Figures and Tables

**Table 1:** Self-concept dimensions in IG and CG at pre-intervention and post-intervention.

Dimensions	Moment	Group		Z	Sig.
		Intervention M (SD)	Comparison M (SD)		
BA—Behavioral adjustment	Pre-intervention	9.68 (2.25)	10.39 (2.41)	8.66	0.01 **
	Post-intervention	10.09 (2.84)	7.26 (4.59)		
ISS—Intellectual and school status	Pre-intervention	7.27 (2.68)	8.30 (3.52)	1.13	0.29
	Post-intervention	7.32 (3.21)	7.17 (3.13)		
PAA—Physical appearance and attributes	Pre-intervention	4.36 (1.71)	5.43 (2.06)	3.00	0.09
	Post-intervention	4.32 (2.12)	4.35 (1.87)		
AC—Anxiety	Pre-intervention	4.05 (2.10)	4.09 (2.10)	4.42	0.04 *
	Post-intervention	3.55 (2.52)	5.13 (.2.01)		
AC—Popularity	Pre-intervention	6.91 (2.41)	7.26 (1.89)	0.22	0.64
	Post-intervention	6.50 (2.77)	6.43 (3.13)		
HS—Happiness and Satisfaction	Pre-intervention	6.36 (1.89)	7.00 (1.57)	5.94	0.02 *
	Post-intervention	6.68 (1.64)	5.39 (3.04)		
AC—Total score	Pre-intervention	38.64 (9.30)	42.48 (11.17)	2.74	0.11
	Post-intervention	38.45 (10.64)	35.74 (15.38)		

* *p* < 0.05; ** *p* < 0.01.

**Table 2:** Emotional and Behavioral Problems in IG and CG at pre-intervention and post-intervention.

Dimensões	Momento	Group		Z	Sig.
		*IG* M (SD)	*CG* M (SD)		
YSR—Internalizing Problems	Pre-intervention	18.35 (8.65)	16.58 (11.55)	3.32	0.08
	Post-intervention	18.09 (9.52)	12.46 (7.03)		
YSR—Externalizing Problems	Pre-intervention	11.70 (6.91)	11.54 (9.34)	0.04	0.84
	Post-intervention	10.09 (7.49)	9.63 (7.89)		
YSR—Total Problems	Pre-intervention	73.04 (23.62)	70.08 (33.92)	2.13	0.13
	Post-intervention	69.78 (26.23)	58.17 (21.85)		

## Data Availability

The data presented in this study are available on request from the corresponding author.
